# Comparative analysis of the nasal effects of two alar cinch suture techniques in LeFort I osteotomy. A retrospective longitudinal study

**DOI:** 10.1007/s00784-026-06755-5

**Published:** 2026-01-30

**Authors:** Martin Orozco-Fernández, Carlos Torres, Juan Pablo Mejía, Alejandro Malaver, Wanderley Augusto Arias-Ortiz, Juan Pablo López

**Affiliations:** 1 Department Oral and maxilofacial surgery, Hospital Universitario Clínica Colombia, Bogotá, Colombia; 2 Oral and maxilofacial surgery program, Universidad El Bosque, Bogotá, Colombia; 3 Hospital Militar Central de Colombia, Bogotá, Colombia; 4 Faculty of Medicine, Research group “Medicina Comunitaria y Salud Colectiva”, Universidad El Bosque, Bogotá, Colombia; 5 Hospital Universitario Fundación Santa Fe de Bogotá, Bogotá, DC Colombia; 6 Unidad de Investigación en Epidemiología Clínica Oral UNIECLO, Universidad El Bosque, Bogotá, DC Colombia

**Keywords:** Le fort i osteotomy, Cinch suture, Nasal width, Alar base width

## Abstract

**Aim:**

The purpose of this study was to compare postoperative nasal morphological changes between two intraoral alar cinch suture techniques in patients undergoing Le Fort I osteotomy.

**Methods:**

A retrospective longitudinal study was conducted with patients undergoing Le Fort I osteotomy, and two types of alar cinch were evaluated. Patients with a history of nasal procedures or cleft lip surgery were excluded. Group 1 received a cinch that passes through the anterior nasal spine, and Group 2 involves only soft tissues. A descriptive analysis was performed, and the normality of variables was assessed using the Shapiro–Wilk test. The repeated measures ANOVA test was then used to evaluate changes over time between the two groups. When the assumption of sphericity was violated, the Mauchly’s test, Greenhouse–Geisser, Huynh–Feldt, and Lower-Bound tests were used to correct for it. Between- and within-subjects effects were analyzed using Type III sums of squares. A significance level of 0.05 was considered.

**Results:**

(*N* = 60) were randomly assigned to two groups (*n* = 30). No significant differences were found in demographic or clinical variables at baseline (*p* > 0.05). A considerable increase in interalar width was seen before and after surgery (*p* < 0.001), with more significant differences in group 1 (*p* = 0.004). Nasal projection and nasolabial angle changed between pre- and postoperative periods (*p* < 0.001 and *p* = 0.003, respectively); however, there was no difference in the type of suture technique used (*p* = 0.474 and *p* = 0.647).

**Conclusions:**

Orthognathic surgery produces significant changes in nasal morphology. However, only the interalar width showed differences between sling techniques, with a greater increase in the group using the sling that passes through the anterior nasal spine. These findings indicate that the choice of sling affects control of the nasal base, while projection and the nasolabial angle depend on maxillary movement.

## Introduction

Although some cases of mandibular prognathism may require isolated mandibular surgery, particularly when cephalometric analysis indicates a predominantly mandibular discrepancy, in clinical practice, many patients present with combined alterations of the maxillomandibular complex. In these scenarios, correction usually involves a Le Fort I osteotomy, alone or in combination with mandibular setback, to restore facial harmony and functional occlusal balance. This procedure results in changes to the soft tissues of the face, particularly affecting the middle third, which reaches stability six months after surgery, as demonstrated by the study conducted by Oh et al. in 2013 [1]. Three-dimensional changes influence soft tissues in the position of the jaws, dependent on movement, with the nose and upper lip being one of the most compromised areas. Therefore, control of changes after Le Fort I osteotomy must be predictable [2]. Nasal cinch as a step in orthognathic surgery has generated controversy due to the lack of consensus on selecting the correct technique. Systematic reviews that combine data from different studies indicate that both the conventional transseptal technique and the modified external skin technique are effective; however, the traditional technique is more consistent in maintaining the width of the nose from the outset [3]. Other clinical trials have examined the classic transseptal technique, along with the modified external technique for moving the upper jaw, such as pushing it up, and found that the transseptal technique provides better control over the width of the alar nose, indicating it offers more substantial support for the stitches [4]. In 2015, Chen et al. also reported a result differing from the above, comparing the classic transseptal technique with the modified lateral technique in a randomized clinical trial and finding no statistically significant differences, thereby leaving an inconsistency in the literature [5]. However, a subsequent study that compared the nasal cinch in LeFort I osteotomies in patients with prognathism with and without asymmetry did show the crucial changes of performing a nasal cinch technique not only controls the width of the alar base but also results in positive changes to the nasolabial angle, highlighting the necessity of this step in maxillary surgery [6]. There is limited comparative evidence on intraoral webbing techniques in Le Fort I osteotomy, which underlines the need for further studies evaluating their effects on nasal changes.

The authors propose that maxillary fixation to the anterior nasal spine could limit anteroposterior nasal displacement, as the three-dimensional relationship between the maxillary bone and the soft-tissue nasal base may influence the transmission of surgical changes. Based on this premise, the main objective of this study was to characterize postoperative nasal changes in adult patients with dentofacial anomalies undergoing maxillary advancement via Le Fort I osteotomy. Specifically, variations in interalar width, nasal projection, and nasolabial angle were evaluated, as well as the potential effect of different intraoral sling techniques used to control the alar base. The authors hypothesize that both the fixation site to the anterior nasal spine and the selected sling technique can differentially influence the magnitude and directionality of nasal changes following maxillary advancement.

## Materials and methods

A retrospective longitudinal study was conducted with a sample derived from the Clínica Colombia, Bogotá, Colombia, and Hospital Militar Central, Bogotá, Colombia. Subjects from the maxillofacial surgery service were considered between May 2019 and november 2024. The study was conducted in accordance with the Declaration of Helsinki of 1964 and its subsequent amendments. The sample was selected according to the inclusion criteria of adult patients with dentofacial anomalies who had an indication for advanced LeFort I osteotomy and met a minimum follow-up period of 6 months. Among the exclusion criteria were patients who required nasal surgery, had a history of cleft lip and palate, or required additional intraoperative soft tissue procedures that could alter the measurements.

The diagnosis of dentofacial anomaly was initially made with plain profile, anteroposterior, and panoramic radiographs for cephalometric studies to arrive at the diagnosis. Subsequently, virtual planning was performed with facial tomography using Dolphin Imaging software (Dolphin Imaging & Management Solutions, Chatsworth, CA, USA). In this study, the alar cinch technique was considered the primary predictor variable, given its potential impact on the transverse, anteroposterior, and aesthetic control of nasal morphology during Le Fort I osteotomy. The evaluated outcomes corresponded to three key parameters of nasal morphology: interalar width, defined as the transverse distance between the lateral alar points; nasal projection, defined as the anteroposterior displacement of the pronasal point relative to a standardized reference plane; and the nasolabial angle, measured as the angular relationship between the columella-subnasal and the upper lip. Additionally, age, sex, and the magnitude of maxillary advancement were included as covariates to control for their potential influence on postoperative variations and to ensure an accurate interpretation of the effects attributable to the evaluated sling technique.

For pre- and postoperative measurements, frontal and profile photographs were taken in a 1:1 ratio. A line was drawn along the most lateral part of the nasal alae to measure the interalar distance, and the linear distance was recorded in millimeters. For nasal projection, the measurement was taken along a perpendicular line drawn from the nasal tip to the Barcelona plane, and the measurement was recorded in millimeters. The nasolabial angle formed by the columella and the philtrum was recorded by taking two lines from the subnasal to the columella and from the subnasal to the upper labial surface. Finally, the measurement was recorded in degrees.

### Surgical technique

**Fig. 1:**
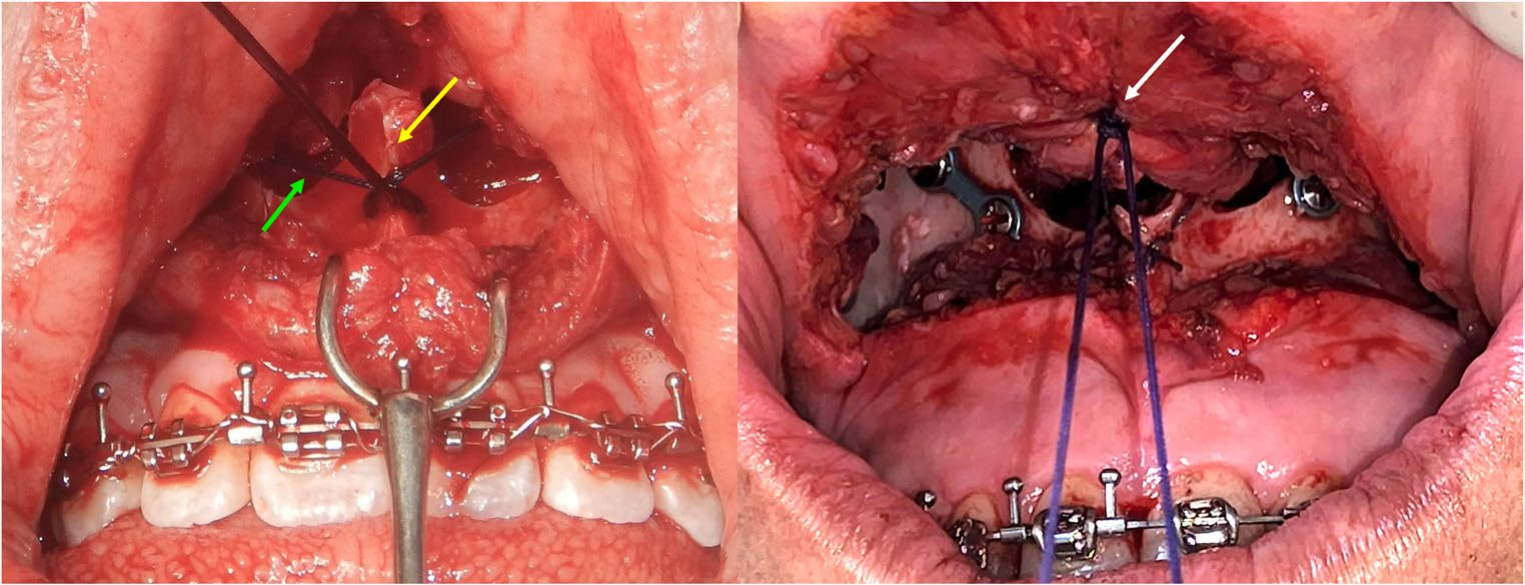
The figure on the left shows the type of strap used in group 1, and the yellow arrow indicates the fixation knot on the nasal spine. The green arrow shows the amount of suture left, which appears to be longer and more prone to loosening. The figure on the right shows the cinch used in group 2, where the knot, indicated by the white arrow, is positioned far from the nasal spine and exhibits no evidence of a long suture, providing a shorter cinch that may be more resistant to loosening

The surgeon performed orthognathic surgery using a conventional high LeFort I technique. The surgeon made the incision at the bottom of the buccal groove, extending from one premolar area to the other. After using ultrasound for the bone cuts, the surgeon checked the separation of the pterygopalatine area and then confirmed that the upper jaw could move properly after it was positioned correctly and secured with plates. The surgeon performed a specific cinch suture, taking into account the group under analysis. In Group 1, the suture was passed through the fibroadipose tissue at the alar base from a lateral to medial direction, passing through a hole created in the nasal spine, and then from medial to lateral direction on the opposite side. In group 2, the suture was threaded through the fibroadipose tissue at the alar base, from the outside to the inside, and then from the inside to the outside on the other side; however, it did not pass through the nasal spine (Fig. 1). Both techniques utilized a non-resorbable monofilament 3 − 0 suture and were then closed in a standard manner.

### Data analysis

Data were analyzed using descriptive statistics to summarize the demographic and clinical characteristics of the population. Quantitative variables were described using measures of central tendency and dispersion, while qualitative variables were summarized using frequencies and percentages. The Shapiro–Wilk test was used to assess the normality of quantitative variables. Differences between groups at two time points were analyzed using a repeated-measures ANOVA. Multivariate results were evaluated using Pillai’s Trace, Wilks’ Lambda, Hotelling’s Trace, and Roy’s Largest Root, all assessed via the F-distribution.

Mauchly’s test was applied to examine the sphericity assumption; when violated, corrections were made using the Greenhouse–Geisser, Huynh–Feldt, and Lower-Bound adjustments. Levene’s test was used to evaluate the homogeneity of variances. The linear trend across time points was also analyzed using the F-statistic to determine significance.

The researchers employed the Type III sum of squares for analyzing between-subjects effects and within-subjects contrasts. A significance level of 0.05 was considered. All statistical analyses and plots were conducted using SPSS version 26 (IBM Corp., Armonk, NY, EE.UU).

## Results

The sample consisted of 60 subjects, equally distributed into two groups, each with 30 subjects. In group 1, the mean age of the participants was 27.7 ± 10.83 [16–47]; in group 2, the mean age was 25.53 ± 9.55 [14–56], with a p-value = 0.43 between groups. Regarding gender in groups 1 and 2, there were 15 women in group 1 and 20 women in group 2. However, there were no statistically significant differences, with a p-value of 0.19 for the comparison of gender between groups. According to the magnitude of maxillary advancement in group 1, the mean advancement was 5.77 ± 1.28, and in group 2, it was 5.5 ± 1.48, with a p-value = 0.315. The presurgical interalar distance in Group 1 had a mean of 36.6 ± 4.02, while Group 2 had a mean of 35.6 ± 2.61, with a p-value of 0.176. In the presurgical nasal projection, the mean of group 1 was 18.51 ± 3.64, and group 2 had an initial mean of 19 ± 3.2, with a p-value = 0.554. Finally, the presurgical nasolabial angle of group 1 had a mean of 91.1 ± 12.45, and group 2 had a mean of 94.5 ± 13.87, with a p-value = 0.193. Taking into account the above, the groups were comparable from the beginning of the study, without presenting statistically significant differences (Table 1).

**Table 1:** Descriptive analysis of the sample

		Type of cinch				
		Group 1		Group 2		P-value
		n	%	n	%	
Gender	Female	15	50	20	-66,60%	0,19*
	Male	15	50	10	33.3	
Diagnostic	Maxillary Retrognathism	27	90	29	96,67	
	Maxillary Prognathism	3	10	0	0	
	Maxillary Retrognathism and vertical excess	0	0	1	3,33	
Dentofacial anomalie	Class III	18	60	15	50	
	Class II	6	2	8	26,67	
	Class III asymetry	6	2	5	16,67	
	Class I asymetry	0	0	2	6,67	
Age	mean SD [IC]	27,7± 10,83	[16-47]	25,53 ± 9,55	[14-56]	0,43 †
Point A (mm)	mean SD [IC]	5,77 ± 1,28	[3-8]	5,5 ± 1,48	[3-9]	0,31 §
Interalar distance	mean SD [IC]	36,8 ± 4,02	[31-45]	35,6 ± 2,61	[30- 40]	0,176 §
Nasal proyection	mean SD [IC]	18,51 ± 3,64	[11.7-27]	19,03 ± 3,2	[11-26]	0,554 †
Nasolabial angle	mean SD [IC]	91,1 ± 12,45	[60-110]	94,53 ± 13,87	[52-114]	0,193 †

Analysis performed using the Chi2 (*)*, Analysis performed using U Mann-Whitney (†)*, *Analysis performed using T student* (§).

Abbreviations: *IC* Interval confidence; *SD* standard deviation

Regarding postoperative measurements by group, the postoperative interalar distance had a mean of 39.5±4.73 in group 1, while the mean of group 2 was 36.97±3.10, with a p-value of 0.18. Regarding the postsurgical nasal projection, group 1 had a mean of 19.81±3.07 and group 2 a mean of 20.7±2.93, and a p-value of 0.258. Finally, regarding the postoperative nasolabial angle, group 1 had a mean of 94 ± 12 and group 2 a mean of 99 ± 12, with a p-value of 0.159. Taking into account the above, the groups were also comparable in the postoperative period (Table 2).

**Table 2:** Comparative post-surgical analysis according to the types of suture cinch

	Type of cinch		Mean difference [CI]	P-value
	Group 1	Group 2		
	mean SD	mean SD		
Interalar distance	39,5±4,73	36,97±3.11	2,533 [0,46-4,61]	0,18
Nasal proyection	19,81±3,07	20,7±2.93	-0,88 [-2,44-0,66]	0,258
Nasolabial angle	94±12	99±12	-4,46 [-10,73-1,80]	0,159

Abbreviations: *IC* Interval confidence; *SD* standard deviation

When performing the repeated measures ANOVA on the interalar distance results, the pre-post multivariate tests showed a statistically significant p-value of < 0.001 in all tests, as well as a p-value of 0.004 for these cinch-type measurements. This indicates that statistically significant differences are evident both in the overall before-and-after averages and between cinch types.

When checking sphericity with Mauchly’s W test, a p-value of 0.001 or less was found, indicating that the sphericity assumption is not met. Therefore, the Greenhouse-Geisser (G-G), Huynh-Feld (H-F), and lower-bound corrections were employed, which showed a p = < 0.001 for the within-subject values and a p-value of less than 0.004 for the cinch type, indicating statistically significant differences between groups and times.

Meanwhile, a test to verify the existence of a linear pattern in the data revealed statistical significance, indicating that the interalar distance value varies over time (*p* < 0.001) and that the type of cinch also varies (*p* = 0.004), suggesting a linear relationship in both cases. Finally, the within-subjects effect reveals marginal significance (*p* = 0.05) between the measurement values​​and cinch type.

**Fig. 2:**
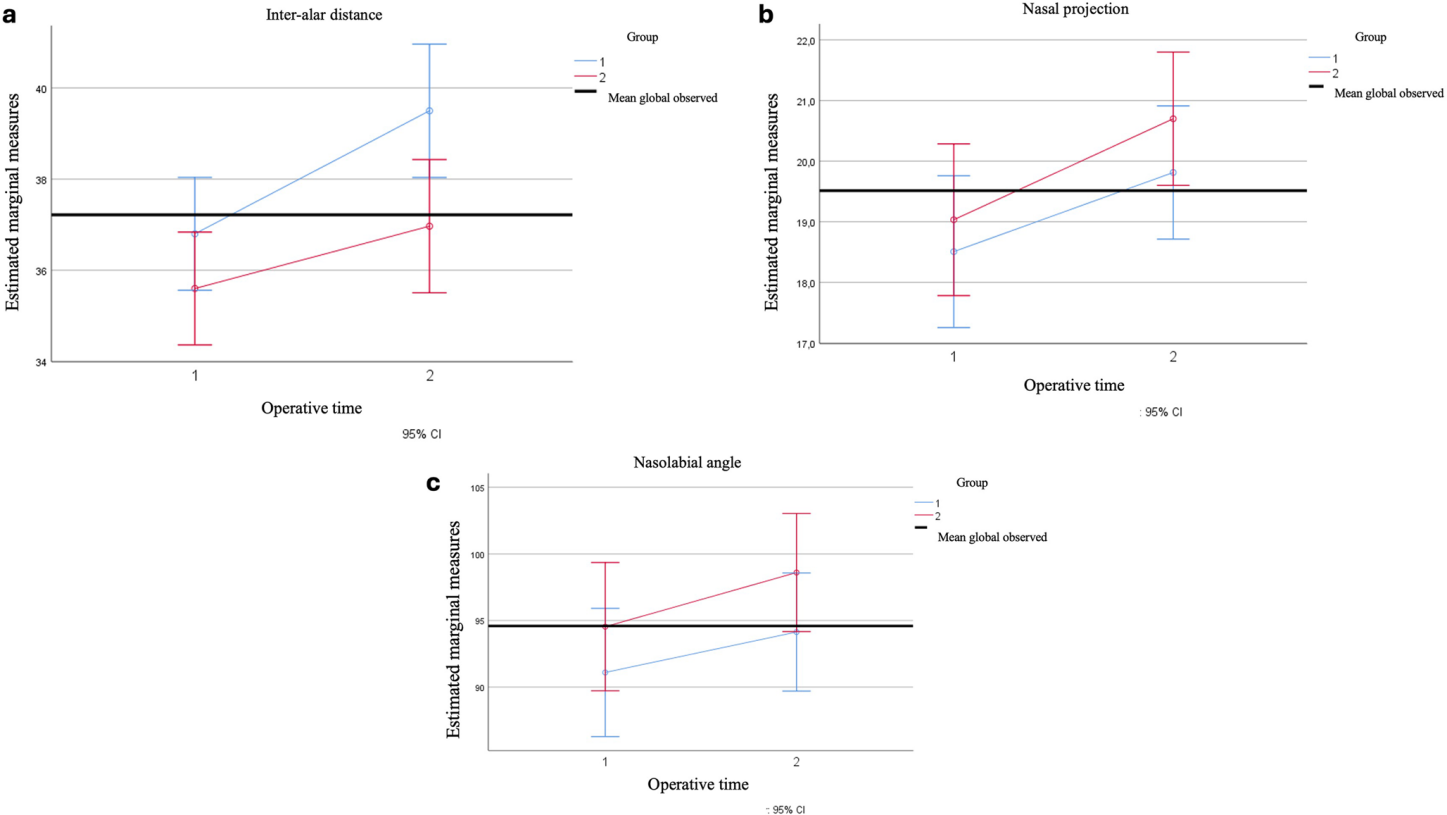
The line graph shows the changes over time 1 and time 2 in the three variables of nasal changes with the two types of nasal strap used

In this sense, significant differences exist in the interalar distance in both measurements (pre- and post-measurement), as well as in the cinch types, with a linear trend over time and cinch type. However, the interaction effect between measurement and cinch type is not statistically significant, indicating that changes in the level occur regardless of the cinch type used (Fig. 2a).

Regarding the projection, statistically significant differences were found in the values over time (*p* < 0.001); however, upon reviewing this difference by type of cinch, no differences were found (*p* = 0.474). For this indicator, the Mauchly W test again presented *p* < 0.001; therefore, the assumption of sphericity is not met. Thus, the G-G, H-F, and lower bound corrections will be used, which confirm the findings of the multivariate with a *p* < 0.001 for the moments but a *p* = 0.474 for the moments by the type of cinch. This finding aligns with the linear pattern, and for the inter-subject effect, it was observed that there were differences between the projection values before and after (*p* < 0.001), but not by the type of cinch (*p* = 0.377). This observation indicates that there is significant linear variation in projection at pre- and post-examination, but it is not affected by the type of cinch used (Fig. 2b).

Finally, statistically significant differences were also found in the analysis of the nasolabial angle for the pre- and post-examination values (*p* = 0.003). However, there were no differences by cinch type (*p* = 0.647). This conclusion was confirmed after verifying the assumption of sphericity (*p* < 0.001) and applying the G-G, H-F, and lower bound corrections. Significance was found in the moment pattern in the linear model with a p-value of 0.003. Regarding the interaction, it was also found that the cinch type does not exhibit statistically significant differences in pre- and post-examination variation, with a p-value of 0.203 (Fig. 2c).

## Discussion

The aim of this study was to evaluate nasal changes following Le Fort I osteotomy and to determine whether different alar sling techniques influence nasal morphology during maxillary advancement. Based on the hypothesis that the sling technique could differentially modify the transverse and anteroposterior stability of nasal morphology, changes in interalar width, nasal projection, and nasolabial angle were analyzed. The results showed a significant increase in all nasal variables after surgery; however, only interalar width showed differences between techniques, while nasal projection and nasolabial angle changed similarly regardless of the type of sling used. These findings suggest that the sling technique plays a more relevant role in the transverse control of the nasal base than in the anteroposterior or angular changes induced by maxillary movement.

Some studies have evaluated the need for alar slings in patients undergoing Le Fort I osteotomy, arguing a possible benefit in the immediate control of the nasal tip and nasolabial angle. However, these studies have shown that these changes tend to disappear over time, suggesting a transient effect of the suture on soft tissues [7]. Unlike our study, these investigations have focused solely on comparing the use versus absence of a nasal sling, without analyzing transverse parameters such as alar base width, which were the only parameters to show significant differences between techniques in our sample. This highlights the importance of comprehensively evaluating nasal morphology, including angular and anteroposterior changes, as well as the transverse behavior of the alar base.

Several studies have indicated that the alar sling can partially reduce the transverse widening caused by Le Fort I osteotomy and maintain some stability over time [8]. However, these studies have been limited to comparing the presence or absence of the sling, without evaluating differences between techniques, which reduces their applicability when the surgeon must select a specific method. Furthermore, these studies have focused exclusively on transverse dimensions, without considering other relevant parameters of nasal morphology, such as projection and the nasolabial angle. In contrast, the present study incorporates these additional components and demonstrates that, although the sling can influence transverse control, anteroposterior and angular changes are primarily determined by maxillary movement. Taken together, these findings offer a more comprehensive and clinically useful view of nasal behavior after Le Fort I osteotomy.

There are modifications to the internal nasal cinch technique, such as its exclusive fixation to soft tissue or its fixation to the anterior nasal spine via bone. Currently, there is no known literature comparing these two techniques in terms of their effects on nasal changes. It is now well accepted that maxillary surgery for the correction of dentofacial anomalies requires special care in the nasal area. For this purpose, the nasal cinch has been studied and compared; however, studies suggest that this surgical step has no significant effect on controlling the widening of the nasal base, as the suture loses tension and progressive widening is observed [9]. In the present study, an increase in nasal measurements was observed, but no differences were found between the groups. However, a widening was noted at 6 months in both groups, possibly due to the loss of suture tension, although it was more pronounced in group 1. On the other hand, comparisons have been made in other studies between intraoral and extraoral techniques, with the conclusion that intraoral techniques have yielded better results [10]. It can be thought that the reason why intraoral techniques present greater control is that they support a greater amount of tissue because the extraoral techniques pass the suture almost exactly through the same area of ​​​​tissue, reducing support.

Given this lack of consensus among studies, systematic reviews and meta-analyses have been conducted. Still, pooled analyses suggest no statistically significant differences between alar strap techniques for controlling nasal width [11]. However, the included studies have evaluated intraoral techniques versus extraoral techniques without differentiating between intraoral techniques. This continues to leave a gap in the literature regarding which technique achieves greater control of nasal width. Additionally, little research has been conducted on other relevant variables, such as nasal projection and nasolabial angle.

Other aspects to consider include the length of the suture after tying, as multiple modifications can be made. Long sutures due to the separation between the nasal wings, with the knot passing through the nasal spine, generate a long suture that may have a greater tendency for the knot to loosen, losing tension early, and being weak in the control of nasal width [12]. This could be an explanation for the statistically significant differences found in the present study, where an increased nasal width was found in group 1, where the knot was fixed to the nasal spine. Additionally, the study by Peacock et al. has highlighted the importance of the piriformis ligament in providing stability and support to the nasolabial muscles, alar cartilages, and all the fibro-alleolar tissue [9]. However, this would be easier to control with intraoral techniques due to the ease of identifying this anatomical structure, which allows controlling the passage of the suture.

One of the advantages of this study is the comparison of two commonly used intraoral techniques, given the scarcity of literature on the subject. Furthermore, this study evaluates nasal width and examines other aspects of the nose, such as the nasolabial angle and nasal projection. The addition of new data contributes to the literature, enabling future studies to provide more solid evidence and allowing researchers to reach more informed conclusions. But, as with all studies of this kind, the sample size may limit the study, even though the statistical tests used reduce error.

The findings of this study are consistent with the literature, which indicates that Le Fort I osteotomy produces significant changes in nasal morphology. However, unlike some previous reports, only the interalar width showed differences between the sling techniques, while the other parameters depended primarily on maxillary movement.

This study has limitations that should be considered, including its retrospective design, sample size, and the inherent subjectivity of photographic measurements—factors that may influence the interpretation of the results.

### Clinical implications

From a clinical perspective, the selection of the cinch technique directly affects control of transverse nasal base widening. However, it does not alter changes in projection or the nasolabial angle, which depend on maxillary advancement. This should be clearly communicated to patients during surgical planning.

### Recommendations for future research

It is recommended to incorporate prospective designs, randomized clinical trials, and larger samples with long-term follow-up that use more objective, ideally three-dimensional, measurement methods to strengthen the available evidence.

## Data Availability

No datasets were generated or analysed during the current study.
